# Activation of Nrf2 signaling: A key molecular mechanism of protection against cardiovascular diseases by natural products

**DOI:** 10.3389/fphar.2022.1057918

**Published:** 2022-12-08

**Authors:** Xiaoyu Wu, Jiajia Wei, Yang Yi, Qihai Gong, Jianmei Gao

**Affiliations:** ^1^ Key Laboratory of Basic Pharmacology of Ministry of Education and Joint International Research Laboratory of Ethnomedicine of Ministry of Education, Zunyi Medical University, Zunyi, China; ^2^ Key Laboratory of Basic Pharmacology of Guizhou Province and School of Pharmacy, Department of Pharmacology, Zunyi Medical University, Zunyi, China

**Keywords:** Nrf2, natural products, cardiovascular diseases, oxidative stress, citespace

## Abstract

Cardiovascular diseases (CVD) are a group of cardiac and vascular disorders including myocardial ischemia, congenital heart disease, heart failure, hypertension, atherosclerosis, peripheral artery disease, rheumatic heart disease, and cardiomyopathies. Despite considerable progress in prophylaxis and treatment options, CVDs remain a leading cause of morbidity and mortality and impose an extremely high socioeconomic burden. Oxidative stress (OS) caused by disequilibrium in the generation of reactive oxygen species plays a crucial role in the pathophysiology of CVDs. Nuclear erythroid 2-related factor 2 (Nrf2), a transcription factor of endogenous antioxidant defense systems against OS, is considered an ideal therapeutic target for management of CVDs. Increasingly, natural products have emerged as a potential source of Nrf2 activators with cardioprotective properties and may therefore provide a novel therapeutic tool for CVD. Here, we present an updated comprehensive summary of naturally occurring products with cardioprotective properties that exert their effects by suppression of OS through activation of Nrf2 signaling, with the aim of providing useful insights for the development of therapeutic strategies exploiting natural products.

## Introduction

Cardiovascular diseases (CVD) are a group of cardiac and vascular disorders including hypertension (HTN), atherosclerosis (AS), myocardial ischemia (MI), heart failure (HF), peripheral artery disease, rheumatic heart disease, and cardiomyopathies (Kolluru et al., 2022). Despite considerable progress in the prophylaxis and treatment of CVDs, associated morbidity and mortality rates remain extremely high, accounting for >32% of all global deaths and presenting a significant socioeconomic burden (Tsao et al., 2022). Importantly, with an aging society worldwide (Liberale et al., 2022) and the recent outbreak of coronavirus disease 2019 (COVID-19), the risk of CVDs continues to increase (Y. Xie et al., 2022), resulting in persistently high incidence and mortality rates. In view of the lack of efficacious therapeutic regimens, the development of novel curative agents to combat CVDs remains an urgent clinical need (X. Li et al., 2021).

While the precise underlying mechanisms are yet to be established, emerging evidence strongly indicates that dysregulation of the redox equilibrium is a hallmark of cardiovascular disease (Xu et al., 2021; Krzemińska et al., 2022). Dysregulation of the redox equilibrium is generally triggered by oxidative stress (OS), defined as a shift in the equilibrium between generation of reactive oxygen species (ROS, such as hydrogen peroxide [H_2_O_2_] and superoxide [O_2_^.-^]) or reactive nitrogen species (RNS, such as nitric oxide [NO^.^]) and their elimination by antioxidants, thereby leading to cell injury (Griendling et al., 2021). ROS/RNS act as a double-edged sword during physiological and pathological processes. Under physiological conditions, ROS/RNS are involved in cellular signal transduction while in the presence of excess ROS/RNS, pathologies leading to CVD are triggered (Panda et al., 2022). Thus, comprehensive understanding of the mechanisms underlying CVDs is essential for the development of novel and efficient therapeutic options for prophylaxis and treatment.

**FIGURE 1:**
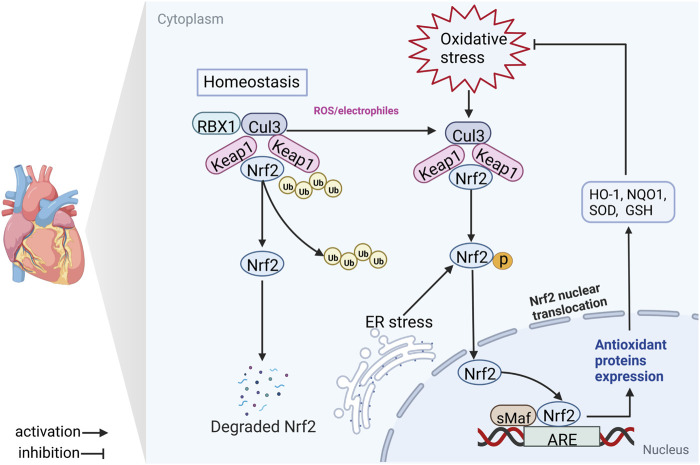
Schematic diagram of the Nrf2/ARE signaling axis. In the majority of conditions, Keap1 binds the Cul3 E3 ligase, forming a Keap1-Cul3-RBX1 E3 ligase complex, which promotes ubiquitination and degradation of Nrf2. Nrf2 is cleaved from the Keap1-Cul3-RBX1 complex and translocases to the nucleus where it heterodimerizes with sMaf and binds ARE in response to antioxidant stress (electrophilic, ROS or ER stress) damage. Nrf2, nuclear factor erythroid 2-related factor 2; Keap1, Kelch-like ECH-associated protein 1; RBX1, RING-box protein 1; Ub, ubiquitin; Cul3, cullin 3; ER stress, endoplasmic reticulum stress; sMaf, small musculoaponeurotic fibrosarcoma; ARE, antioxidant response element; GSH, glutathione; NQO1, NADPH quinone oxidoreductase 1; SOD, superoxide dismutase; HO-1, heme oxygenase 1.

Intriguingly, accumulating evidence suggests that cells generally offset the negative effects of ROS/RNS *via* activation of nuclear factor E2-related factor 2 (Nrf2; gene name Nfe2l2) and impaired activation of Nrf2 is associated with CVD progression (Gutiérrez-Cuevas et al., 2022). Nrf2 is a “Cap’n’Collar” transcription factor with a b-ZIP domain, which governs multiple genes encoding proteins with cytoprotective effects, such as nicotinamide adenine dinucleotide phosphate (NADPH) quinone dehydrogenase 1 (NQO1), heme oxygenase 1 (HO-1), isocitrate dehydrogenase 1 (IDH1), and glutathione (GSH) (Zhu et al., 2022). Under physiological conditions, Nrf2 is controlled by the negative regulator Kelch-like ECH-associated protein 1 (Keap1) and undergoes successive ubiquitination and proteasomal degradation (Torrente & DeNicola, 2022). However, in response to OS, Keap1 is oxidized and inactivated, promoting Nrf2 translocation from the cytoplasm to nucleus where it binds to the antioxidant response element (ARE) as a heterodimer and facilitates transcription of target genes (e.g., HO-1, NQO1, glutathione peroxidase [GSH-Px], superoxide dismutase [SOD], catalase [CAT]) (Huang et al., 2020). Bach-1 and sMaf proteins are dissociated, promoting dimerization of Nrf2 with sMaf and gene transcription (Q. Zhang L. M. et al., 2021) (Figure 1). Therefore, Nrf2 presents a potential target for the development of novel candidate agents that confer protective effects against CVDs through suppression of OS. Bioactive phytochemicals and other natural products are extensively used to prevent and treat multiple disorders owing to their high efficacy and safety profiles. Several natural products have achieved encouraging outcomes for CVDs and may serve as valuable resources for novel drug discovery (D. Yao et al., 2022).

In this review, we provide a comprehensive overview of the protective role of the Nrf2 signaling pathway in CVDs. The natural products or derivatives with beneficial properties against CVDs through activation of Nrf2 signaling are summarized, with the aim of providing a reference for subsequent research on CVD management.

## Role of Nrf2 signaling in CVDs

Earlier studies have confirmed a vital role of OS in CVD progression and the protective effect of Nrf2 signaling (Andreadou et al., 2021). In view of the activity of Nrf2 as a critical regulator of antioxidant defense, the premise that Nrf2 protects against OS-related cardiovascular disease is feasible.

### Nrf2 and arterial HTN

Earlier studies reported distinct physiological equilibrium between ROS/RNS and antioxidants necessary for multiple cellular functions. Overproduction of ROS/RNS impairs vascular cell function along with reduced availability and vasoconstriction of NO^.^, in turn, eliciting HTN (Griendling et al., 2021; Krzemińska et al., 2022; Tanase et al., 2022). While the precise role of Nrf2 in HTN remains to be established, several associations among Nrf2 and ROS/RNS have been detected in HTN animal models (Tanase et al., 2022). Nrf2 is crucial in the regulation of blood pressure (BP) in multiple ways. One of the underlying mechanisms is overexpression of HO-1, which has been shown to decrease BP in a spontaneously hypertensive rat (SHR) animal model (Gao et al., 2017; X. Tan et al., 2021). HO-1 degrades heme to produce carbon monoxide (CO) (Liao et al., 2021), a new type of gaseous signal molecule that can regulate the vascular response through vasoconstrictor suppression *via* activation of soluble guanylate cyclase and generating cyclic guanosine monophosphate (cGMP) (Álvarez-Maestro et al., 2021). In addition, biliverdin, another product of HO-1 degradation, is transformed to the powerful antioxidant, bilirubin, and further stimulates HO-1 activity for maintaining vascular homeostasis (Cuadrado et al., 2019). In the SHR model, the levels of Nrf2 and its target antioxidant enzymes are reduced, which results in decreased antioxidant activity, increased OS and vascular disruption (Lopes et al., 2015; Ye et al., 2022). Conversely, activation of Nrf2 effectively sustains the redox equilibrium by reducing OS, increases endothelial-dependent vasodilation and decreases BP in the vasculature of the SHR model (Meephat et al., 2021). Similarly, HO-1 expression effectively reverses OS injury in mice with arterial HTN and vascular dysfunction caused by environmental stress, such as noise exposure (Bayo Jimenez et al., 2021). The Nrf2-induced vasoprotective effect on HTN may present another protective mechanism (Tanase et al., 2022). Selective Nrf2 gene deletion in the rostral ventrolateral medulla.

Interestingly, studies to date support a dual role of Nrf2 in diabetes-related HTN. In diabetic Akita mice, genetic deletion of Nrf2 or pharmacological inhibition of Nrf2 attenuated HTN through upregulation of intrarenal angiotensin converting enzyme 2 and angiotensin 1-7 receptors. The opposite effect of Nrf2 activation may be due to Nrf2-mediated stimulation of intrarenal renin-angiotensin system, by which chronic hyperglycemia induces HTN and renal injury in diabetes (S. Zhao et al., 2018). Although the precise role of Nrf2 in diabetes-related HTN remains controversial, numerous preclinical studies suggest that activation of Nrf2 signaling offers a promising strategy for amelioration of vascular OS and endothelial dysfunction during HTN.

#### Nrf2 and AS

AS is a primary contributor to CVDs, such as MI and HF (Liberale et al., 2021), predominantly through permeation and accumulation of low-density lipoproteins (LDL) into the arterial intima where they are subjected to oxidation (T. X. Zhao & Mallat, 2019; Roy et al., 2022). Oxidation of LDL (ox-LDL) promotes endothelial lesions and activation of foam cells, leading to the formation of fatty streaks in carotid atherosclerotic walls (Alonso-Piñeiro et al., 2021). OS modulated by ROS/RNS therefore plays a key role in the process of AS. At the blood vessel level, multiple endogenous enzymes are considered primary sources of ROS/RNS, including lipoxygenase, NADPH oxidase (NOX), myeloperoxidases, xanthine oxidase (XO) and uncoupled endothelial NO^.^ synthase (eNOS) (Tejero et al., 2019). Enzymes of the NOX family convert O_2_ to O_2_^.-^, which reacts with NO^.^, generating peroxynitrite (ONOO^−^), thereby inducing endothelial cell damage (Hahner et al., 2020; Ogboo et al., 2022). In addition, uncoupled eNOS contributes to the generation of O_2_^.-^ and ONOO^−^, leading to impairment of endothelial function and initiation of AS (Das et al., 2021).

Various antioxidant systems, including SOD, CAT and GPx, within the carotid atherosclerotic wall play a crucial role in counteracting ROS/RNS generation (Alonso-Piñeiro et al., 2021). During this process, Nrf2 acts as a major modulator of antioxidant defense through not only increasing antioxidant enzyme expression but also mediating NADPH oxidase activity (Q. M. Chen, 2021). Nrf2 is closely associated with AS and reported to play an antagonistic role in its development. For instance, in high-fat high-cholesterol diet (HFD)-treated LDL receptor-deficient (LDLR^−/−^) mice, specific Nrf2 deficiency in bone marrow-derived cells exacerbated early AS (Ruotsalainen et al., 2013).

Recent studies have identified Nrf2 as a key regulator of endothelial miRNA expression under oxidized phospholipid stimulation, which contributes to decreased proliferation of injured vascular endothelial cells (Linna-Kuosmanen et al., 2021). In contrast to these protective effects, Nrf2 has also been shown to exert pro-atherogenic effects. For instance, Nrf2 deficiency protected against AS in ApoE^−/−^ mice (Barajas et al., 2011; Ruotsalainen et al., 2019). Moreover, loss of Nrf2 resulted in increased uptake of modified LDL in thioglycollate-elicited peritoneal macrophages and attenuated the atherosclerotic plaque burden, proliferation, and migration of vascular smooth muscle cells (Caselli et al., 2021; H. Li et al., 2022) reported that in patients, higher plasma HO-1 levels are associated with lower cholesterol and a more diffuse but mainly non-obstructive coronary AS, confirming a potential role of the Nrf2/HO-1 pathway as a protective feedback mechanism.

### Nrf2 and MI or reperfusion

The definition of MI is based on evidence of myocyte death on account of extended ischemia associated with occlusion of the coronary artery (Algoet et al., 2022). To rescue cardiac tissue from ischemic damage, reperfusion is accomplished by coronary artery bypass grafting surgery or non-surgical percutaneous coronary intervention (Q. M. Chen, 2021). Although these therapies are beneficial in ameliorating symptoms, reducing infarct size, and sustaining ventricle function, reperfusion triggers further myocyte death, with consequent augmentation of myocardial infarct size (Zeng et al., 2021). Accumulating evidence indicates that OS is a major cause of poor prognosis in MI or reperfusion, leading to myocardial cell death and reduced systolic-diastolic function (Rajesh et al., 2010; Lei et al., 2018). Interestingly, earlier experimental evidence supports the utility of Nrf2 as a lead target for drug development to further improve treatment outcomes for MI and reperfusion (Z. Jiang et al., 2020; Q. Zhang et al., 2022).

In H_2_O_2_-treated H9c2 cells, knockdown of Nrf2 was recently shown to induce a decrease in the levels of antioxidant enzymes (GSH, SOD, CAT) in association with increased OS (Z. Zhang et al., 2022). Similarly, in H9c2 cells exposed to hypoxia/reoxygenation (H/R)-induced injury, Nrf2 silencing promoted H/R-stimulated OS damage (W. Jiang et al., 2021; M. Zhang et al., 2021). Moreover, genetic and pharmacological activation of Nrf2 effectively protects against ischemic heart injury in animal models while Nrf2 deficiency exacerbates HF after myocardial infarction (Ashrafian et al., 2012; Cao et al., 2015). These findings collectively highlight the importance of Nrf2 in mitigating ischemic heart disease and support the application of effective Nrf2 stimulators as potential treatment agents for MI reperfusion injury (MIRI). In the mouse model, a key role of Nrf2 in protecting the heart has been widely demonstrated. Earlier studies indicated that cardiomyopathy is exacerbated in Nrf2 deficient (Nrf2^−/−^) mice after PM2.5 insult (Ge et al., 2020; J. Liu et al., 2022). Augmentation of MI, cardiac insufficiency and senescence in Nrf2^−/−^ mice were induced by left coronary artery ligation (Luo et al., 2020). In cardiac-specific Nrf2 transgenic mice exposed to isoproterenol (ISO), enhanced Nrf2-dependent antioxidant defense suppresses OS and prevents pathological cardiac remodeling (Shanmugam et al., 2019). Induction of Nrf2 antioxidant signaling delivers protection against MIRI by suppressing mitochondrial ROS production and improving bioenergetics *in vitro* and *in vivo* (Duan et al., 2017; Y. Q. Li et al., 2022; H. Xu et al., 2022).

#### Nrf2 and HF

Patients recovering from MI or reperfusion are at considerably higher risk for progression of HF in subsequent years (Mauro et al., 2022; Salerno et al., 2022). An imbalance between oxygen radical synthesis and elimination by antioxidant defense mechanisms leads to macromolecular damage and disruption of cellular redox signaling, which affects cardiac structure and function (Leyane et al., 2022). Continuous OS contributes to myocyte death or cardiac dysfunction (X. Xu et al., 2022). Increasing evidence supports a crucial role of Nrf2 in the process of HF.

In an HF mouse model, Nrf2 was expressed at low levels in the rostral ventrolateral medulla and its overexpression led to significantly reduced sympathetic nerve activity and improved arterial function (Ma et al., 2019). Excessive OS in obesity and HF with preserved ejection fraction induced by a high-fructose/high-fat Western diet is associated with reduced phosphorylation of Nrf2 (Hayden & Bostick, 2019). Furthermore, Nrf2 depletion leads to increased cardiac damage and accelerated HF after ischemic injury (Strom & Chen, 2017; Gutiérrez-Cuevas et al., 2022). Recently, Wu et al. (2021) demonstrated that an antagonist of Nrf2 increased the ER OS response of cardiomyocytes in HF rats. Activation of Nrf2 successfully alleviated diastolic dysfunction and reduced NO *in vivo* (Yoon et al., 2021) and induced a significant increase in NQO1 expression, conferring beneficial antioxidant effects and reducing afterload in the myocardium of patients with HF (A. Zhang et al., 2018). The collective results suggest that activation of Nrf2 is effective in prevention of cardiomyocyte injury in HF.

#### Nrf2 and other CVDs

The transcription factor Nrf2 exerts beneficial effects against cardiovascular injury (for example, doxorubicin (DOX)-induced cardiotoxicity, diabetic cardiomyopathy (DCM) and cardiorenal syndrome (CRS)) by maintaining the redox equilibrium. Downregulation of Nrf2 and HO-1 expression was observed in an earlier mouse model of DOX-induced chronic cardiotoxicity (X. Li et al., 2022). More recent studies on cultured rat cardiomyocytes showed that overexpression of Nrf2 ameliorates DOX-induced myocardial oxidative damage, mitochondrial dysfunction and cardiac myocyte death *via* enhancing anti-oxidative activity (Y. Wang et al., 2022) whereas its knockdown exacerbates OS, mitochondrial dysfunction and cardiac myocyte death (W. B. Zhang et al., 2021; J. Li et al., 2022). DOX-induced cardiotoxicity was amplified in Nrf2^−/−^ mice, along with increased myocardial OS and cardiomyocyte necrosis (W. B. Zhang et al., 2021). Accumulating evidence over the last few years has demonstrated a critical role of the Nrf2/ARE signaling axis in preventing hyperglycemia-induced OS in diabetic heart (Syed et al., 2021). In a diabetic mouse model, the Nrf2^−/−^ phenotype was shown to induce cardiac remodeling and dysfunction (Gao et al., 2018; Fang et al., 2022). Moreover, silencing of Nrf2 increased MDA and decreased superoxide dismutase levels in glucolipotoxicity-induced H9c2 cells (L. Wang et al., 2022). Conversely, activation of the Nrf2 pathway significantly increased cardiomyocyte viability, reduced ROS formation and enhanced antioxidant enzyme activity in a Type 2 diabetic rat model (X. Wu et al., 2022). Diseases of the kidney and heart often occur in parallel, eventually leading to CRS, whereby acute or chronic dysfunction of one organ induces the same disorder in the other (Patel et al., 2022). In the 5/6 nephrectomy-induced CRS mouse model, the intensity of ROS expression was markedly increased and Nrf2 was significantly reduced in kidney and heart tissue (Yang et al., 2019; Wang et al., 2020). Similarly, the increase in OS due to Nrf2 depletion aggravated cardiac dysfunction in the CRS model (L. Xu et al., 2022). Additionally, aging is one of the most critical risk factors for the development of CVDs and their complications (Kloska et al., 2019). There are several studies demonstrate that Nrf2 exerts beneficial effect on vascular function in aging CVDs. In aged rhesus macaques (Macaca mulatta, age: ≥20 years) animal model, the level of ROS in carotid arteries was significantly increased in comparison with young macaques (age: ∼10 years); Whereas, age-related OS did not activate or induce Nrf2 and its downstream target genes (e.g. NQO1, GCLC, and HMOX1). Interestingly, in cultured vascular smooth muscle cells that derived from aged macaques or young macaques, H_2_O_2_ combined with high glucose-induced Nrf2 activity and Nrf2-driven gene expressions were blunted in vascular smooth muscle cells from aged macaques than those of young macaques (Ungvari et al., 2011a). Moreover, in 24-month-old aged mice, Nrf2 dysfunction can impair cellular stress recovery and increase OS, thereby accelerating vascular aging (Fulop et al., 2018). Nrf2 deficiency contributes to the increased OS in cardiac muscle and vasculature during aging (Ungvari et al., 2011b; Kubben et al., 2016). Of note, in Nrf2^−/−^ mice, the effects of cardiovascular risk factors (e.g. obesity) were enhanced due to Nrf2 deficiency accelerates cellular senescence (R. Wang et al., 2017; Tarantini et al., 2018).

## Protective effects of natural products on CVDs

Comprehensive studies have verified that natural products from herbal medicines exert excellent protective effects against CVDs through activation of the Nrf2 pathway. In the following sections, recent developments in studies on the beneficial effects of natural products against CVDs associated with Nrf2 are reviewed.

### Natural plant extracts

In the ISO-induced MI model, *Rhaponticum carthamoides* (Willd.) Iljin and Myrrh essential oils induce upregulation of Nrf2 and HO-1 and protect the oxidative status of cardiac myocytes by increasing the activities of cardiac antioxidants, such as GSH, SOD and CAT (Younis & Mohamed, 2021; Zheng et al., 2022). In addition, an aqueous extract of *Amauroderma rugosum* (AR) has been shown to significantly promote Nrf2/HO-1 signaling and reduce OS, mitochondrial dysfunction and apoptosis in Dox-treated H9c2 cells or mice. Notably, knockdown of Nrf2 abolished the protective effect of AR in these cells (J. Li et al., 2022) (Table 1).

**TABLE 1:** Protective effects of herbal medicinal extracts in vascular endothelial cells.

Herbal medicine	Model	Dose/Concentration	Effects	Related molecular targets		References
				Upregulation	Downregulation	
Rha	SD rats with ISO-induced MI; CoCl2-induced H9c2	40, 80, and 160 mg/kg; 100, 200, 400 μg/ml	Reducing BP; inhibiting OS; disruption of energy metabolism	SIRT6, Nrf2, MDA, SOD, ROS		Zheng et al. (2022)
MEO	SD rats with ISO-induced MI	50 mg/kg	Inhibiting OS; reducing cell apoptosis; inhibiting inflammation	GSH, SOD, CAT	MDA	Younis and Mohamed, (2021)
AR	Dox-induced Cardiotoxicity in mice or H9c2 cells	250 mg/kg; 0.125–2 mg/ml	Decreasing OS, mitochondrial dysfunction and apoptosis	p–Akt/Akt, p–mTOR/mTOR, Nrf2, HO–1	Cleaved PARP/PARP, Cleaved caspase-9/caspase-9, Cleaved caspase-3/caspase-3, Bax/Bcl-2, ROS, Keap1, LDH	Li et al. (2022)

#### Flavonoids

Phytoconstituents, such as flavonoids, terpenoids and phenols with anti-OS abilities, have significant therapeutic potential against cardiovascular damage. Flavonoids possess highly beneficial anti-oxidant, anti-inflammatory, anti-viral, anti-diabetic, anti-cancer, neuroprotective, and cardioprotective properties (Naeem et al., 2022). In recent years, the potential of flavonoids against diseases related to AS, MI and HF has been clinically confirmed. Baicalin is a plant-derived flavonoid with cardioprotective effects that increases hypoxia-stimulated H9c2 activity and expression of Nrf2 and HO-1 proteins (H. Yu et al., 2019). In addition, baicalin reduces ROS and inflammation *via* Nrf2 activation in human umbilical vein vascular endothelial cells (HUVECs) stimulated with high glucose or mice with streptozotocin (STZ)-induced diabetes. However, reported that the protective effects of baicalin are almost abolished in HUVECs transduced with shRNA against Nrf2 (G. Chen et al., 2018).

*Arthrinium sp.*, 2,3,4,6,8-pentahydroxy-1-methylxanthene and Arthon C are novel flavonoids from deep-sea fungi, which effectively scavenge ROS and enhance nuclear translocation of Nrf2 and its downstream antioxidant gene, HO-1. Knockdown of Nrf2 abolishes these effects and exacerbates ox LDL-induced oxidative damage in HUVECs (Hou et al., 2021). Anthocyanin in plants promotes Nrf2 activation and release in a concentration-dependent manner, leading to increased HO-1 and NQO1 expression in HUVECs under hyperoxia (Cimino et al., 2013; Herrera-Bravo et al., 2022). Cyanidin-3-O-glucoside, another type of anthocyanin, induces nuclear localization of Nrf2 and activation of cellular antioxidant and cytoprotective genes in palmitate or TNF-α-induced HUVECs (Speciale et al., 2013; Fratantonio et al., 2015). Additionally, irigenin is reported to elevate cell viability and balance the OS status of HUVECs after exposure to angiotensin II (Ang II) through activation of Nrf2 signaling and, conversely, Nrf2 deficiency abrogates the protective effects of irigenin (Q. Zhang M. et al., 2021). Moreover, hesperidin is a naturally occurring flavonone, which presents in citrus fruits and has been shown to protect against cardiac OS in aged rat through activates Nrf2 and then maintains the redox status (Elavarasan et al., 2012).

Diosmetin is a citrus flavonoid that improves endothelial dysfunction, suppresses over activity of sympathetic nerve-mediated vasoconstriction and upregulates Nrf2 and HO-1 proteins in Nω-nitro-l-arginine methyl ester l-NAME-induced HTN rats (Meephat et al., 2021). Recent study further indicates that diosmetin improves cardiomyocyte hypertrophy and inhibits cardiac OS through increasing protein expression of Nrf2 and Keap1 in aortic banding-induced pressure overload mouse models or phenylephrine (PE)-induced neonatal rat ventricular myocytes. However, blockade of Nrf2 activation offsets the diosmetin-mediated protective effects against PE *in vitro* (Guo et al., 2022). Moreover, xanthohumol, a major prenylated chalcone found in hops, exerts antioxidant effects to inhibit vascular calcification in rat induced by vitamin D3 plus nicotine through enhanced nuclear translocation of Nrf2 and HO-1 (Liou et al., 2020). Interestingly, however, in H9c2 cells treated with Fe-SP and RSL3 or ischemia/reperfusion (I/R)-induced rat heart, xanthohumol induced a decrease in the Nrf2 level to protect the myocardium against I/R injury-induced ferroptosis (Lin et al., 2022).

In an LAD-induced MI model, isoliquiritigenin (ISL) or icariin significantly reduced the myocardial infarction area, improved cardiac function, inhibited ROS and MDA production, suppressed SOD and GPx consumption through increased nuclear Nrf2 and cytosolic HO-1 levels and reduced OS after AMI (Sai et al., 2022; D. Yao et al., 2022). Furthermore, in mice or rats with STZ-induced diabetes mellitus or high glucose-cultured H9c2, ISL attenuated inflammation and OS and protected against aortic injury through upregulation of Nrf2 and HO-1 (X. Gu et al., 2020; Alzahrani et al., 2021).

Pinocembrin, a flavonoids compound from honey, propolis and *Boesenbergia pandurata*, exhibits diverse biological activities reported to enhance ROS uptake and activate Nrf2 in LAD-induced HF or DOX-induced cardiotoxicity (J. Gu et al., 2021). Interestingly, specific Nrf2 inhibitors significantly reversed the antioxidant effects of pinocembrin *in vitro* (X. Chen et al., 2021). Cardamonin, a flavone compound naturally residing in multiple herbs, such as *Alpinia katsumadai*, *Ginkgo biloba*, and *Carya cathayensis Sarg*, under conditions of LPS-induced HF or DOX-induced cardiotoxicity, effectively inhibited cardiac mechanical dysfunction and OS through promoting nuclear translocation of Nrf2 along with increasing HO-1 and antioxidant defense, which were reversed upon intervention with a Nrf2 inhibitor (Qi et al., 2020; Y. Tan et al., 2021). Kaempferol, another natural flavonoid widely distributed in plants, possesses multiple pharmacological properties, including anti-inflammatory, anti-oxidant and ant-apoptotic activities (Sharma et al., 2019). Protective cardiovascular effects of kaempferol through activation of the Nrf2 pathway have been demonstrated in numerous animal models, including ISO-induced HF in diabetic rats (L. Zhang et al., 2019), HFD-induced AS in APOE^−/−^ mice (Feng et al., 2021), STZ-induced DCM in mice (X. Chen et al., 2018), and LAD induced MIRI (D. Wang et al., 2017) (Table 2).

**TABLE 2:** Protective effects of flavonoids on vascular endothelial cells.

Monomer	Model	Dose/Concentration	Effects	Related molecular targets		References
				Upregulation	Downregulation	
Baicalin	Hypoxia-induced injure in H9c2; STZ-induced diabetic in mice; high glucose-induced oxidative damage in HUVECs	75 μM; 50 mg/kg; 50 μM	Enhancing cell viability; reducing cell apoptosis; decreasing OS	HIF1a, BNIP3, Nrf2, HO-1		Chen et al. (2018); Yu et al. (2019)
*Arthrinium sp.*, 2,3,4,6,8-pentahydroxy-1-methylxanthene; Arthon C	ox-LDL-induced oxidative injury in HUVECs	1M, 5M, 50M	Inhibiting apoptosis and adhesion factor expression	Nrf2, HO-1	ROS	Hou et al. (2021)
Anthocyanin	HUVECs or hyperoxia-induced damage in HUVECs	100 μg/ml	Decreasing OS	Nrf2, HO-1, NQO1		Fratantonio et al. (2015); Speciale et al. (2013)
Cyanidin-3-O-glucoside	Palmitate-induced endothelial dysfunction or TNF-α-induced damage in HUVECs	20, 40 μM	Improving; intracellular redox status; inhibiting inflammation	Nrf2	NF-kB, Bach1	(Fratantonio et al., 2015) (Speciale et al., 2013)
Irigenin	Ang II-induced OS damage in HUVECs	2.5, 5, 10, 20, 40 μmol/L	Enhancing HUVEC viability; reducing; apoptosis and OS	Nrf2, HO-1, NQO1	Bax, Bcl, Caspase-3, Cleaved Caspase-3	Zhang et al. (2021b)
Hesperidin	aged Wistar rats	100 mg/kg	Protecting cardiac tissue; reducing OS	Nrf2, SOD, CAT, GPx, GR, G6PD	MDA, Keap1	Elavarasan et al. (2012)
Diosmetin	Nω-nitro-l-arginine methyl ester l-NAME-induced HTN in SD rats	20, 40 mg/kg	Improving endothelial dysfunction; suppressing sympathetic nerves	Nrf2, HO-1, p-JNK	IL-6, p-NF-κB	Meephat et al. (2021)
	Aortic banding-induced pressure overload model in mice or phenylephrine-induced cardiac hypertrophy in neonatal rat ventricular myocytes	40 mg/kg or 10, 50 μm	Reducing cardiac hypertrophy and dysfunction	Nrf2, NQO1, HO-1, SOD2, p-mTOR/mTOR, p-AKT/AKT, P-PI3K/PI3K	LC3I/II, p62, Beclin1, ATG7	Guo et al. (2022)
Xanthohumol	vitamin D3 plus nicotine-induced vascular calcification in SD rats	20 mg/L	Reducing calcium content and alkaline phosphatase activity; decreasing superoxide and ROS generation	Nrf2, HO-1	Keap1 BMP-2, Runx2, *α*-SMA, SM22α	Liou et al. (2020)
	Langendorff- induced MIRI in rat or Fe-SP and RSL3 (0.1 μM)-induced injure in H9c2	5, 10 μM or 10, 20 μM	Reducing infarct size; chelating iron; inhibiting; ROS and lipid peroxidation	GPX4	Nrf2, ACSL4	Lin et al. (2022)
Isoliquiritigenin	LAD-induced MI in C57BL/6 mice	100 mg/kg	Reducing myocardial infarction area; improving cardiac function; reducing OS	Nrf2, HO-1, SOD, GPx	NF-κB, ROS MDA	Yao et al. (2022)
	STZ-induced injury in mice or SD rats or high glucose-induced oxidative damage in H9c2 cells	10, 20 mg/kg; 10 μM	Reducing inflammation and OS	Nrf2, Keap-1, HO-1	IL-10, IL-6, TNF-α, Caspase-3	Alzahrani et al. (2021); Gu et al. (2020)
Icariin	LAD-induced MI in C57BL/6 mice	60 mg/kg	Reducing inflammation; minimizing myocardial cell injury and immune response; reducing myocardial apoptosis	Nrf2, HO-1	Bax, Bcl, Caspase-3, Cleaved Caspase-3, TNF-α, IL-1β, INF-γ, IL-17	Sai et al. (2022)
Pinocembrin	LAD-induced MI in rat; ISO-induced injury in H9c2; DOX-induced cardiotoxicity in mice	5 mg/kg; 25 μM	Reducing apoptosis; facilitating angiogenesis	Nrf2, HO-1, SOD	p53, Bax, Bcl, Caspase-3, Cleaved Caspase-3, ROS, MDA	Chen et al. (2021); J. Gu et al., 2021)
Cardamonin	LPS induced OS in mice or cardiomyocytes; DOX (5 μM)-induced cardiotoxicity in H9C2 or HL-1 cells	20 mg/kg, 10 μM; 25, 50, 100 μM	Inhibiting cell OS and apoptosis	Nrf2, HO-1, catalase, GPx1, SOD1	Caspase-3 Bax Bcl-2, NF-κB; IκBα, IL-1β, IL-6, TNF-α	Qi et al. (2020); Tan et al. (2021)
Kaempferol	ISO and STZ-induced HF in diabetic rats; HFD-induced AS in APOE^−/−^ mice; ox-LDL- induced oxidative injury in HAECs; STZ-induced diabetic mice; LAD-induced MI in rats	20 mg/kg; 50, 100 mg/kg; 5, 10 or 20 μM	Decreasing fasting blood glucose and glycosylated hemoglobin levels; increasing serum insulin levels; inhibiting endothelial cell apoptosis	Nrf2, HO-1	NF-κB, Caspase-3 Bax, Bcl-2, PI3K, Akt, GSK-3β, troponin-I, LDH, ROS, TNF-α, IL-6	Chen et al. (2018); Feng et al., 2021; D. Wang et al., 2017; Zhang et al. (2019)

#### Terpenoids

Terpenoids produced by plants are specific metabolites with significant biological activity that show considerable promise as therapeutic compounds. Terpenoids have been shown to exert protective effects against CVDs through activation of the Nrf2 pathway. For instance, kirenol, a terpenoid derived from *Herba Siegesbeckia,* attenuates benzo[a]pyrene-induced AS in HUVECs *via* activation of Nrf2. Notably, however, Nrf2 deficiency largely abolishes the protective effects of kirenol on HUVEC cells following OS challenge (Rajendran et al., 2021). Tanshinone I, a primary component of *Salvia miltiorrhiza*, confers a variety of favorable pharmacological activities, including cardiovascular protection. In MI/R animal model, tert-butyl hydroperoxide-stimulated H9c2 cells (Zhuo et al., 2021) or DOX-induced cardiotoxicity model (Q. Jiang et al., 2022), tanshinone I significantly attenuates OS through activation of Nrf2 to promote antioxidant-related protein expression. However, its protective effect was abolished in Nrf2^−/−^ mice (Y. T. Wu et al., 2021). In addition, dihydrotanshinone-I (DT) has been shown to ameliorate MIRI and H/R injury with maintenance of redox homeostasis by activating Nrf2. However, DT fails to improve the final infarct size and subsequent cardiac remodeling in Nrf2^−/−^ mice after MIRI. Moreover, DT-induced beneficial effects are abolished in Nrf2-depleted cardiomyocytes, further supporting a pivotal role of Nrf2 in therapeutic efficacy of DT (Zeng et al., 2021).

In mice with LAD-induced MI or diabetic cardiomyopathy, andrographolide effectively reduces OS and enhances antioxidant stress capacity through activation of Nrf2 (Liang et al., 2018). Conversely, a specific Nrf2 inhibitor or silencing of Nrf2 substantially counteracts the protective effects of andrographolide in H9c2 (S. Xie et al., 2020). Ruscogenin, a naturally occurring steroidal sapogenin, induces a significant decrease in infarct size and ameliorates biochemical indicators and cardiac pathological changes after MI in mice through activation of the Keap1/Nrf2/HO-1 signaling pathway (Fu et al., 2022). Nootkatone is a naturally occurring bioactive sesquiterpene shown to attenuate myocardial OS in ISO-induced MI in by activating the PI3K/Nrf2/Akt cascade (Meeran et al., 2021). Moreover, astragaloside IV has been shown to exert potent cardioprotective effects in various animal models, including abdominal aortic constriction (AB)-induced HF, LAD-induced HF and MIRI, through activation of Nrf2/HO-1 signaling (M. Jiang et al., 2019; Nie et al., 2019; Sui et al., 2020). Astragaloside IV additionally prevents ox-LDL-induced HUVEC injury through mediation of Nrf2/HO-1 signaling (Z. Zhu et al., 2019) (Table 3).

**TABLE 3:** Protective effects of terpenoids on vascular endothelial cells.

Monomers	Model	Dose/Concentration	Effects	Related molecular targets		References
				Upregulation	Downregulation	
Kirenol	benzo[a]pyrene-induced OS in HUVECs	25 μmol	Attenuating OS; enhancing antioxidant capacity	Nrf2, PI3K, AKT, pPI3K, pAKT, Nrf2, HO-1, NQO-1	Bcl2, Bax, caspase-3, 4-HNE, MDA	Rajendran et al. (2021)
Tanshinone I	Tert-butyl hydroperoxide induced OS in H9c2; DOX- induced cardiotoxicity in mice; ISO-induced myocardial damage model	1 μM; 10 mg/kg	Attenuating necroptosis; reducing OS	Akt, Nrf2, NQO-1, HO-1	p-RIP1, p-RIP3, p-MLKL, ROS, MDA, IL-6, TNF-α	Jiang et al. (2022); Wu et al. (2021); Zhuo et al. (2021)
Dihydrotanshinone-I	LAD-induced MI in C57BL/6 mice; H/R induced injure in HL-1 cells	5 mg/kg; 0.1, 1, 5 μmol/L	Reducing myocardial infarct size; maintaining redox homeostasis	Nrf2, NQO-1, HO-1, PKB, GSK-3β	PKC-δ, Fyn	Zeng et al. (2021)
Andrographolide	LAD-induced MI in C57/BL6; hypoxia-induced OS in H9c2; STZ-induced diabetic mice	25 mg/kg; 12.5, 25, 50 or 100 µM	Inhibiting inflammation cardiac fibrosis; remodeling after infarction; enhancing antioxidant stress capacity	Nrf2, HO-1 SOD2; HIF1α	TGFβ, smad3, IκBα, P65, Gp91, Keap-1	Liang et al. (2018); Xie et al. (2020)
Ruscogenin	LAD-induced MI in mice; hypoxia-induced OS in H9c2	0.375, 0.75, 1.5 mg/kg; 0.1, 1, 10 μM	Decreasing infarct size; ameliorating biochemical indicators; inhibiting ferroptosis	GPX4, Nrf2, HO-1, BCAT1, BCAT2	Keap1, ACSL4, FTL	Fu et al. (2022)
Nootkatone	ISO-induced MI in Wistar rats	10 mg/kg	Restoring cardiac function and reducing OS; inhibiting inflammation; reducing cardiomyocyte apoptosis	PI3K/Akt GSH NrF2/Keap1/HO-1	TLR4-NFκB/MAPK	Meeran et al. (2021)
Astragaloside IV	AB-induced MI in rats; Ang II-induced injure in H9c2; ox-LDL- induced OS in HUVECs; LAD-induced MI in SD rats	40, 80 mg·/kg; 25, 50, or 100 μM; 10, 20, 50 μM	Attenuating cardiac hypertrophy; improving left ventricular function and structure; reducing OS	Nrf2, HO-1, NQO-1, SOD-2, Txn-1	NPA, TNF-α, IL-6, ROS	Jiang et al. (2019); Nie et al. (2019); Sui et al. (2020); Zhu et al. (2019)

#### Alkaloids

Sulforaphane (SFN) is a natural plant compound found in many cruciferous vegetables such as broccoli, cabbage, cauliflower and kale. SFN attenuates OS and cell death by reducing ROS *via* activation of Nrf2 in several *in vitro* models, including Ang II-induced HUVEC injury (M. Zhang et al., 2020), TGF-*β*_1_-induced activation of rat cardiac fibroblasts (Fix et al., 2019) and DOX-induced cardiotoxicity in H9c2 cells (Li et al., 2015). Furthermore, SFN protects cardiomyocytes from OS by activating Nrf2 in mouse models in several *in vivo* animal models, including Ang II-induced cardiomyopathy in mice (Xin et al., 2018), HFD/STZ-induced diabetic cardiomyopathy in mice (J. Gu et al., 2017; Y. Sun et al., 2020; X. Wang et al., 2022) and intermittent hypoxia-induced cardiomyopathy in mice (Zhou et al., 2018). Moreover, SFN also prevents age-associated cardiac and muscular dysfunction through activation of Nrf2 (Angulo et al., 2019; Bose et al., 2020). Remarkably, there are currently more than 20 ongoing clinical trials of SFN for treating multiple disorders such as diabetes, autism, breast cancer, chronic obstructive pulmonary disease, asthma, non-alcoholic fatty acid. Notably, both the sprout extract and highly purified SFN show excellent safety profile in clinic (Cuadrado et al., 2019). Berbamine, a natural compound of berberis, alleviates LAD-induced MIRI by inhibiting OS through promoting Nrf2 nuclear translocation. A Nrf2-specific inhibitor has been shown to counteract the antioxidant effects of berbamine (C. Xu et al., 2022). Recent studies indicate that songorine protects cardiac function in LPS-treated mice and rescues cardiomyocytes from LPS-induced endotoxin insult through activation of Nrf2/ARE and Nrf1 signaling cascades (Y. Li et al., 2021). In addition, sinomenine ameliorates cardiac hypertrophy in ISO-induced HF through activating the Nrf2/ARE signaling pathway. However, Nrf2 inhibitors abolish the protective effect of sinomenine on HF (Yuan et al., 2021) (Table 4).

**TABLE 4:** Protective effects of alkaloids on vascular endothelial cells.

Monomer	Model	Dose/Concentration	Effects	Related molecular targets		References
				Upregulation	Downregulation	
Sulforaphane	Ang II-induced HUVEC injury or cardiomyopathy in mice	2 μM; 0.5 mg/kg	Attenuating OS and cell death	Nrf2, SOD, CAT, GSH, NQO1	ROS	Xin et al. (2018); Zhang et al. (2020)
	TGF-β1-induced rat cardiac fibroblasts	0, 10, 20 μM		HMOX 1, SOD1, Nrf2	MMP9	Fix et al. (2019)
	DOX-induced cardiotoxicity in H9c2 cells	10 μM	Reducing ROS levels attenuating OS	Nrf2, Keap1, HO-1, ARE	ROS	Li et al. (2015)
	HFD/STZ-induced diabetic cardiomyopathy in mice	0.5 mg/kg	Improving cardiac dysfunction, OS, inflammation, fibrosis, and hypertrophy	Nrf2, HO-1, NQO1 GSH, CAT	IL-6	Gu et al. (2017); Sun et al. (2020); Wang et al. (2022)
	Intermittent hypoxia-induced cardiomyopathy in mice	0.5 mg/kg	Increasing antioxidant capacity	Nrf2, SOD2, NQO1, MT		Zhou et al. (2018)
	21- to 22-month-old mice; vascular tissues from 20-month-old SD rats	442.5 mg/kg; 10 μM	Restoring mitochondrial function, cardiac function, exercise capacity, glucose tolerance	Nrf2, CAT, SOD1, SOD2, HO-1		Angulo et al. (2019); Bose et al. (2020)
Berbamine Songorine	LAD and reperfusion induced MIRI in C57/BL6 mice	10 mg/kg	Reducing myocardial infarct size; inhibiting OS; apoptosis; alleviating MIRI and the mitochondrial state	AMPK, Nrf2, HO-1, NQO-1 SOD	LDH, ROS, MDA	Xu et al. (2022)
	LPS-induced sepsis in C57BL/6 mice	50 mg/kg	Improving cardiac function; inhibiting OS	Nrf2, ARE, NRF1, PGC-1α, SOD2, TFAM	ROS	Li et al. (2021)
Sinomenine	Ang II-induced injure in H9C2, or ISO-induced MI in mice	50, 100 μM; 80 mg/kg	Relieving OS; reducing apoptosis	Nrf2, ARE, HO-1	ROS, MDA, Caspase-3, Bax	Yuan et al. (2021)

#### Glucosides

Sweroside, a secoiridoid glucoside extracted from *Swertia pseudochinensis* Hara, has a wide range of pharmacological properties, including antioxidant and anti-inflammatory effects. In H9c2 with H/R-induced injury and a rat model with Langendorff-induced MIRI, sweroside effectively improved cardiac function and reduced OS through promoting nuclear translocation of Nrf2. These beneficial effects were blocked by a Nrf2 inhibitor (J. Li et al., 2021). Pubescenoside A has been reported to exert cardioprotective effects with anti-OS properties in multiple experimental models, including LAD-induced MIRI, LPS/ATP-mediated RAW264.7 macrophages, OGD/R-induced H9c2 cells and primary cardiomyocytes, through activation of the Nrf2 pathway, which are almost offset in Nrf2^−/−^ mice. Further mechanistic studies have revealed selective covalent binding of pubescenoside A to conserved cysteine residues, specifically, cysteine 77 in the BTB domain and cysteine 434 in the Kelch domain of Keap1, with consequent inhibition of Nrf2 ubiquitination and activation of antioxidant enzymes (Cheng et al., 2021) (Table 5).

**TABLE 5:** Protective effects of glucosides on vascular endothelial cells.

Monomer	Model	Dose/Concentration	Effects	Related molecular targets		References
				Upregulation	Downregulation	
Sweroside	Langendorff- induced MI in Wistar rats; H/R-induced OS in H9c2	25, 50, 100 mg/kg; 50 µM	Reducing infarct size; improving cardiac function; partially inhibiting OS and pyroptosis	Nrf2, HO-1, NQO1	Keap1 NLPR3, cleaved caspase-1, IL-1β	Li et al. (2021)
Pubescenoside A	LAD-induced MI in rats; LPS plus ATP-induced injure in RAW264.7 cells; OGD/R-induced injure in H9c2 and primary cardiomyocyte cells	30 mg/kg; 10, 30 μM	Reducing infarct size; restoring cardiac function and decreasing inflammation and OS	Nrf2, HO-1, NQO1	Keap1, NLPR3, cleaved-caspase1, IL-1β, ACS, Caspase1	Cheng et al. (2021)

##### Other products

In addition to the above compounds, numerous other natural products have been shown to exert protective effects on CVDs through activation of Nrf2 signaling. Withaferin A, a natural phytochemical derived from the plant *Withania somnifera*, time- and concentration-dependently induces HO-1 expression in HUVECs and EA.hy926 *via* upregulation and increased nuclear translocation of Nrf2 (Heyninck et al., 2016). Similarly, in endothelial cell line (EA.hy926) with tert-butyl hydroperoxide-induced injury originating from fusion of human umbilical vein endothelial and A549 human lung carcinoma cells, Z-ligustilide attenuates atherosclerotic plaque formation and enhances antioxidant enzyme activity through activation of Nrf2 signaling (Y. Zhu et al., 2019).

In LAD- and HFD-induced ApoE^−/−^ or HFD-induced LDLR^−/−^ mice, resveratrol and Z-ligustilide block the development of AS *via* inducing Nrf2 activity (Seo et al., 2019). Furthermore, resveratrol mitigates HTN in SHR through stimulation of Nrf2 activity (Javkhedkar et al., 2015; Y. Zhu et al., 2019). Of note, anti-inflammatory and antioxidant effects of resveratrol are ongoing in phase 3 clinical trial. Curcumin is a known natural compound with multiple bioactivities, including antitumor, antioxidant and anti-inflammatory activities. Study has shown that curcumin effectively not only improves the histological changes in the myocardial tissues of diabetic rats, but also reduces generation of ROS through activation of the Nrf2 (Xiang et al., 2020; Wei et al., 2022). Furthermore, by modifying the Cys151 residue of Keap1, curcumin inhibits the ability of the Cullin3-Rbx1 E3 ubiquitin ligase complex to ubiquitinate against Nrf2, thereby potentially altering the conformation of the complex, saturating the binding capacity of Keap1 to Nrf2 and facilitating the nuclear translocation of newly synthesized Nrf2 (Shin et al., 2020). Most importantly, supplementation with resveratrol or curcumin do not cause cardiovascular risk as evidenced by ongoing clinical trials (Vors et al., 2018). In mice with LAD-induced MIRI, histochrome, panaxatriol saponin or lithospermic acid exert cardioprotective effects through upregulation of Nrf2 and HO-1 proteins. However, the beneficial effects of these compounds are almost abolished under conditions of Nrf2 deficiency (Hwang et al., 2021; M. Zhang et al., 2021; H. Yao et al., 2022). Zingerone attenuates aortic banding-induced cardiac remodeling and cardiac hypertrophy, along with reducing OS through mediating Nrf2/ARE activation. Consistent with earlier findings, Nrf2 deficiency counteracts the cardioprotective effects of zingerone *in vivo* (C. Liu et al., 2019). Additionally, procyanidins effectively decrease ROS and MDA levels and increase SOD activity in PM2.5-induced rats through upregulation of protein expression of Nrf2 and its downstream antioxidant genes NQO1 and HO-1 (L. M. Zhang et al., 2021). Ellagic acid, a naturally occurring phenolic constituent, derives from fruits and nuts effectively improves hypochlorous acid-induced endothelial dysfunction in HAECs and protects against HFD-induced atherosclerosis in ApoE^−/−^ mice through activation of Nrf2. Whereas, these protective effects of Ellagic acid are substantially offset in Nrf2^−/−^ mice (Ding et al., 2014) (Table 6).

**TABLE 6:** Protective effects of other monomers from herbal medicines on vascular endothelial cells.

Monomer	Model	Dose/Concentration	Effects	Related molecular targets		References
				Upregulation downregulation		
Withaferin A	HUVECs and EA.hy926	1 µM	Increasing antioxidant enzyme activity	HO-1, Nrf2	Keap1	Heyninck et al. (2016)
Z-Ligustilide	HFD-induced AS in LDLR^−/−^ mice; tert-butyl hydroperoxide-induced OS injury in EA.hy926 cells	20 mg/kg; 100 μM	Suppressing AS progression; alleviating lipid peroxidation; increasing antioxidant enzyme activity	Nrf2, ARE, HO-1, NQO1, CAT, SOD1, SOD2, GCLC, GCLM, GR, GS, GPX4, GRX1, GRX2, PRX1, PRX4, PRX6, TR1, TR2, TRX	Keap1	Zhu et al. (2019)
Resveratrol	LAD and high-fat Paigen diet-fed-induced AS in ApoE^−/−^ mice	20 mg/kg	Suppressing inflammation; reducing atherogenic responses	Nrf2, FERM-kinase, cleaved FAK ARE	TNF-α, IL-1β, ICAM-1	Seo et al. (2019)
	SHR	50 mg/kg	Increasing antioxidant capacity	Nrf2, GST, SOD		Javkhedkar et al. (2015)
Curcumin	HFD and STZ-induced diabetic rats; STZ-induced diabetic rabbit	20 mg/kg; 300 mg/kg	Reducing the histological changes in the myocardial tissues	SOD, Nrf2, ARE	MDA	Wei et al. (2022); Xiang et al. (2020)
Panaxatriol saponin	LAD-induced I/R in rat; H_2_O_2_ (200 μM)-induced OS in H9c2 and primary cardiomyocytes	25, 50, 100 mg/kg	Ameliorating mitochondrial OS and cardiomyocyte apoptosis	Nrf2, HO-1	Keap1 Cleaved Caspase-3	Yao et al. (2022)
Histochrome	LAD and reperfusion-induced MIRI in SD rats	1 mg/kg	Reducing infarct size; reversing arrhythmia; reducing OS	Nrf2, Slc7a11, Hmox1, Txnrd1, NQO1, GPx4, GSH, GPx	Keap1	Hwang et al. (2021)
Lithospermic acid	LAD and reperfusion -induced MIRI in C57BL/6 mice; hypoxia and reoxygenation-induced injure in H9C2	50 mg/kg; 100 μM	reducing cardiac damage; ameliorating OS and cardiomyocyte apoptosis	eNOS, iNOS, nNOS, SOD2, Nrf2, HO-1, AMPK	Keap1, Cleaved caspase-3, Bax, Bcl-2	Zhang et al. (2021)
Zingerone	aortic banding surger-induced MI in C57/B6J mice; ISO-induced injure in neonatal rat cardiomyocytes	10, 20 mg/kg; 50, 250 μmol/L	Suppressing cardiac hypertrophy, fibrosis, OS and inflammation	Nrf2, HO-1, SOD, p-eNOS, NO	Keap1, GP-91, TNF-α, IL-1β, IL-6	Liu et al. (2019)
Procyanidins	PM2.5-induced injure in SD rats and vascular smooth muscle cells	50, 100, 200 mg/kg; 20 μM	Attenuating or OS inhibiting apoptosis	Nrf2, NQO1, HO-1, SOD	ROS, MDA	Zhang et al. (2021)
Ellagic acid	HFD-induced atherosclerosis in ApoE^−/−^ mice; hypochlorous acid-induced HAECs	30 mg/kg	Improving endothelium-dependent relaxation, attenuating endothelial dysfunction	Nrf2, NOS, HO-1		Ding et al. (2014)

## Molecular mechanisms of cardioprotective natural products targeting the Nrf2 signaling pathway

**FIGURE 2:**
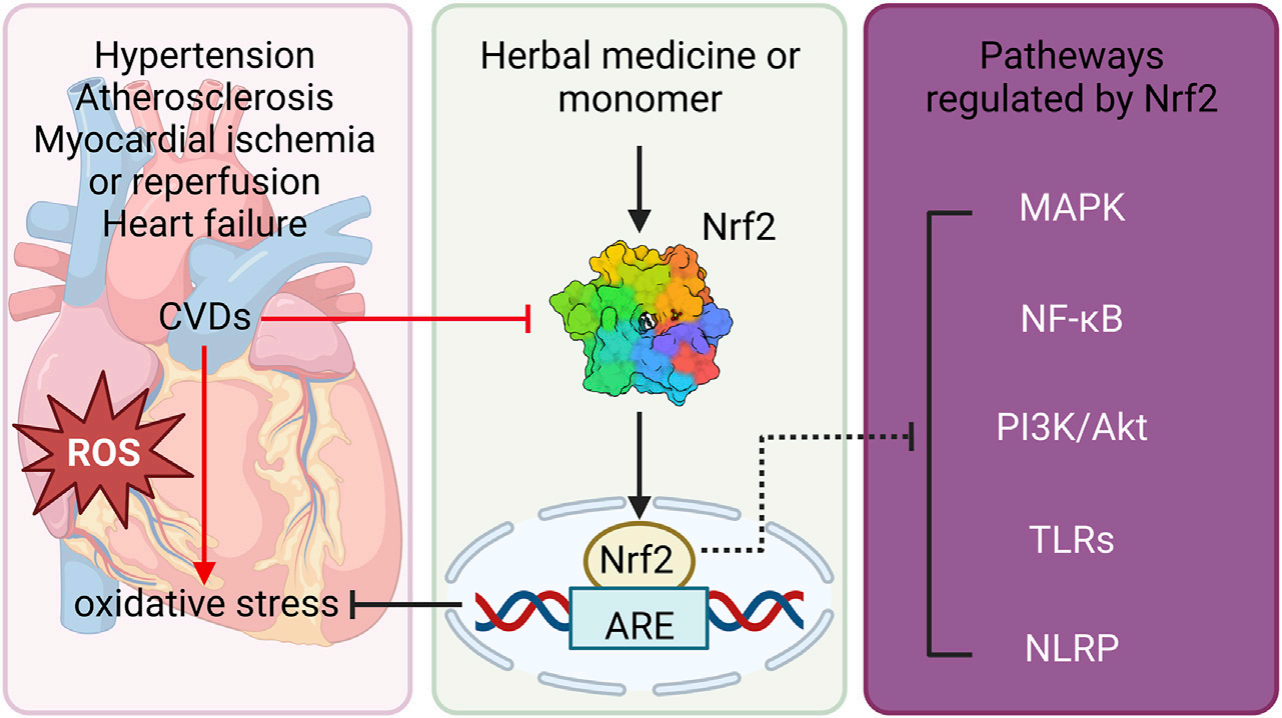
Schematic diagram of natural product-mediated activation of Nrf2 against CVDs. Natural products protect against CVDs including hypertension, atherosclerosis, myocardial ischemia, heart failure *via* reducing OS. Furthermore, the beneficial effects of natural products on CVDs, at least partly, through activation of Nrf2 and its downstream ARE, as well as modulation of other signaling pathways including MAPK, NF-κB, PI3K/Akt, TLRs and NLRP signalings. Nrf2, nuclear erythroid 2-related factor 2; CVDs, cardiovascular diseases; OS, oxidative stress; ARE, antioxidant response element; MAPK, mitogen-activated protein kinase; NF-κB, nuclear factor kappa-light-chain-enhancer of activated B cells; PI3K, phosphatidylinositol-3-kinase; AKT, protein kinase B; TLRs, toll-like receptors; NLRP, NOD-like receptors.

The cardioprotective role of natural products that target the Nrf2 pathway has been widely investigated. There are multiple mechanisms that are involved in activating Nrf2, including interaction with cysteine residues on Keap1, disruption of Nrf2/Keap1 interaction and activation of protein kinases (Figure 2). Keap1 is a cysteine rich adaptor protein, of which Cys151, Cys273 and Cys288 play essential roles in inhibiting the activation of Nrf2 signaling (Saito et al., 2016). Studies have revealed that SFN, rutin and curcumin can inhibit the ability of the Cullin3-Rbx1 E3 ubiquitin ligase complex *via* modifying Keap1 Cys151 residue, resulting in Nrf2 activation to protect against myocardial injury in mice (Takaya et al., 2012; Sthijns et al., 2017; Shin et al., 2020). Similarly, data of molecular docking indicates that the BTB domains in Keap1-Nrf2 complex directly bound the cyanidin-3-glucoside (−6.9 kcal/mol), malvidin-3-glucoside (−6.6 kcal/mol) and peonidin-3-glucoside (−6.6 kcal/mol), which are the major active compound of glycosylated anthocyanins (Herrera-Bravo et al., 2022). Apart from that, a number of bioactive compounds including withaferin A, xanthohumol and betanin can modify cysteine residues, specifically Cys151, Cys273 and Cys288, through interaction with their sulfhydryl groups, allowing the cleavage of the Keap1-Nrf2 interaction inducing antioxidant proteins and phase II detoxification enzymes (Yao et al., 2015; Heyninck et al., 2016; Silva et al., 2022). Emerging evidence demonstrates that several natural product compounds exert cardioprotective effects by disrupting the Nrf2/Keap1 interaction and promoting Nrf2 nuclear translocation. For instance, baicalein and myricetin directily bind Keap1-Nrf2 complex and reduces the steady-state level of Keap1 by increasing its ubiquitination and modification without dissociation from Nrf2, which leads to the disengagement of Nrf2 from ubiquitination (Qin et al., 2013). *α*-Linolenic acid has been found to protect against DOX-induced cardiotoxicity by promoting the degradation of Keap1 and thus facilitating nuclear translocation of Nrf2 *via* directly binds Keap1-Nrf2 complex (X. Yu et al., 2013). Moreover, protein kinases such as ERK, GSK3β, MAPK, and PI3K/AKT can mediate Nrf2 phosphorylation which enhance Nrf2 stability, thereby promoting nuclear Nrf2 translocation and transactivation activity. Recent studies have reported that Baicalein increased the phosphorylation level of MEK1/2, AKT and JNK1/2, and stabilized Nrf2 protein by inhibiting the ubiquitination and proteasomal turnover of Nrf2 (Qin et al., 2014). Genistein and baicalin can activate the Nrf2 pathway to alleviate oxidative injury *via* the activation of the ERK1/2 signaling pathways (Zhai et al., 2013; Shi et al., 2018). In addition, ginsenoside-Rb3 activated the NRF2 signaling pathway through PERK, which can protect cardiomyocytes against H/R injury (J. Sun et al., 2019). These findings suggest that natural products might be directly bind to Keap1-Nrf2 complex or indirect activation by facilitating or inhibiting other factors to confer the cardioprotection.

## Challenges and considerations

Currently, there are still remain challenges and considerations, including pharmacodynamic evaluation and safety profile of Nrf2. Since Nrf2 possesses a very short half-life (15–30 min), the therapeutic benefit of optimal curative dosing regimen needs to be reflected by indirect indicators of its activation in the various damage organs. To solve this question, assessment of drug distribution and gene expression signatures of NRF2 in accessible tissues or cells with a view to that the Nrf2 transcriptomic signature in those cells predicts local engagement in other tissues. In addition, the levels of urinary and its metabolites have been utilized as indicators of pharmacodynamic effect modulated through Nrf2 to avoid exposing to hazardous environmental chemicals. Recent studies reveal that the pharmacodynamics of Nrf2 activators are observed for longer periods of time than the Nrf2 levels and they do not correspond to the concentrations of plasma drug. Therefore, TBE-31, a Nrf2 activator, has a half-life of 10 h in skin and plasma of mice; while, NQO1 is still evident in the murine skin for at day 3 after the last administration of Nrf2 activator. Thus, a model of “indirect pharmacodynamic response” might be a more reasonable approach for assessing the pharmacodynamics of Nrf2 activator towards clinical applications.

The concentration and distribution of phytochemical Nrf2 activators, as well as their solubility also play crucial roles in the extent of NRF2 activation, and eventually leading to the ideal long-lasting pharmacodynamic actions. Often, several phytochemicals have poor bioavailability (e.g. curcumin, resveratrol and baicalin), so much that the systemic concentrations reached are seldom close to the physiological concentrations required for scavenging free radicals. In fact, most studies have used supra-physiological concentrations of these compounds which is very difficult to achieve with normal diet consumption (Y. T. Wu et al., 2021; P. Chen et al., 2022). Thus, optimization of clinically promising drug exposure needs to analyze dose-dependent gene mediation by a putative Nrf2 activator. Moreover, advanced drug delivery systems that achieve maximal therapeutic efficacy and optimal drug dosage may be a logical approach to conquer poor bioavailability of phytochemicals.

Although Nrf2 activator confers cardioprotection against CVDs, it is important to investigate how significant is the risk posed by promoting levels of Nrf2 beyond a safe-threshold. Recent reports have been revealed that somatic mutations in NFE2L2 or Keap1 or collaborate with the associated oncogenic pathways elicit over activation of Nrf2 and accelerate carcinogenesis. Preclinical proof-of-concept studies demonstrated that due to determine the potential cancer risk, dosing of specific Nrf2 activators need to be extremely carefully monitored. Thus, pharmacological activation of Nrf2 to treat CVDs requires not beyond the safe therapeutic window.

## Frontiers and hotspots

**FIGURE 3:**
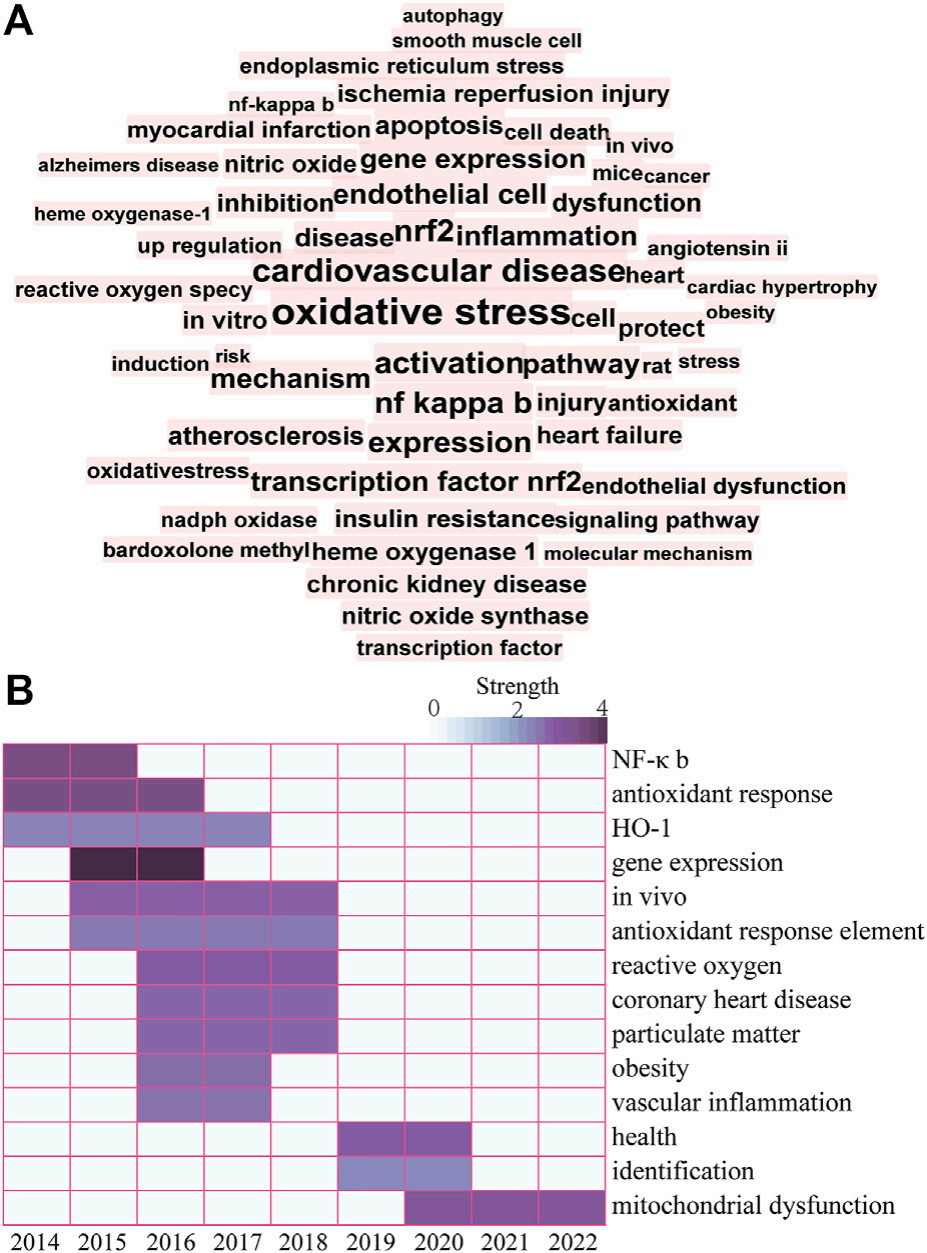
**(A)** Keyword co-occurrence knowledge map. A larger and more centered font corresponds to the number of times a keyword appears. **(B)** Top 14 keywords with the strongest citation bursts. The color depth represents the strength value of keywords with the strongest citation bursts. Darker color corresponds to a larger strength value.

Keyword visualization analysis of the research literature in this area was conducted using CiteSpace software (Figure 3). In brief, the following search strategy was used: TS = “Nrf2” and “Cardiovascular Diseases”. Studies published from the inception of the database to 2022 were retrieved. All valid data were imported to CiteSpace 6.1.R2 Basic and de-duplicated for subsequent visual analysis. Factors used for previous research on the CiteSpace setup were applied as follows: look back years (=5), link retaining factor (=3), e for top N (e = 1), years per slice (1), time span (2014–2022), links selection criteria (g-index, k = 25; strength: cosine, scope: within slices) and pruning (pathfinder-pruning sliced networks) (Y. Zhang et al., 2022). Keywords with frequency >15 were labeled and those with the top 14 strongest bursts identified. Visualization of keywords additionally showed that research hotspots on Nrf2-mediated CVDs in recent years have mainly focused on OS, CVDs, Nrf2, NF-κB, endothelial dysfunction, inflammation, and apoptosis. A map based on the top 14 keywords with the strongest citation bursts was generated, which showed the shift in research hotspots over time. The significant keywords in the early stages (2014–2017) included NF-κB, antioxidant response, HO-1, and gene expression. Over recent years (2019–2022), ARE, ROS, particulate matter, obesity, vascular inflammation and mitochondrial dysfunction have been the main research hotspots. The collective findings validate the beneficial effects of Nrf2 against CVDs, supporting the development of naturally occurring Nrf2 agonists as promising therapeutic approach.

## Conclusion and perspectives

OS plays a vital role in CVD progression and thus presents a key therapeutic target. Cells possess inherent antioxidant systems to overcome the toxic effects of OS, such as the Nrf2 signal transduction pathway. Multiple preclinical experiments support targeting of Nrf2 as an option for OS management. Pharmacological regulators that stimulate the Nrf2 antioxidant defense system may thus offer an effective treatment strategy for OS-associated CVDs. Notwithstanding, in this review, we summarized and updated the potential naturally occurring products with cardioprotective properties on CVDs that exert their effects by inhibition of OS through activation of Nrf2, there are still several pending issues need to be addressed. Firstly, elaborately designed, large cohort clinical trials of naturally occurring Nrf2 activators to ascertain their adverse effects in humans are required in the future. Secondly, the detailed mechanisms of Nrf2 activators against CVDs should be explored in depth. In fact, the ongoing clinical trials will definitely offer momentous advances in answering these pending issues.
